# F/OH ratio in a rare fluorine-poor blue topaz from Padre Paraíso (Minas Gerais, Brazil) to unravel topaz’s ambient of formation

**DOI:** 10.1038/s41598-021-82045-2

**Published:** 2021-01-29

**Authors:** N. Precisvalle, A. Martucci, L. Gigli, J. R. Plaisier, T. C. Hansen, A. G. Nobre, C. Bonadiman

**Affiliations:** 1 Physics and Earth Sciences Department, University of Ferrara, Via Saragat 1, 44122 Ferrara, Italy; 2 Elettra - Sincrotrone Trieste, ss 14, km 163.5, 34149 Basovizza, Trieste Italy; 3 Institut Laue-Langevin (ILL4-138), 71 avenue des Martyrs, 38000 Grenoble, France; 4 Geosciences Institute, University of São Paulo Rua do Lago, 562 - Butantã, São Paulo, 05508-080 Brazil

**Keywords:** Mineralogy, Geochemistry

## Abstract

Topaz [Al_2_SiO_4_(F,OH)_2_] is one of the main fluorine-bearing silicates occurring in environments where variably acidic (F)/aqueous (OH) fluids saturate the silicate system. In this work we fully characterized blue topaz from Padre Paraíso (Minas Gerais, Brazil) by means of in situ synchrotron X-Ray and neutron powder diffraction measurements (temperature range 298–1273 K) combined with EDS microanalyses. Understanding the role of OH/F substitution in topaz is important in order to determine the hydrophilicity and the exchange reactions of fluorine by hydroxyl groups, and ultimately to characterize the environmental redox conditions (H_2_O/F) required for mineral formation. The fluorine content estimated from neutron diffraction data is ~ 1.03 a.f.u (10.34 wt%), in agreement with the chemical data (on average 10.0 wt%). The X_OH_ [OH/(OH + F)] (0.484) is close to the maximum X_OH_ value (0.5), and represents the OH- richest topaz composition so far analysed in the Minas Gerais district. Topaz crystallinity and fluorine content sharply decrease at 1170 K, while mullite phase starts growing. On the basis of this behaviour, we suggest that this temperature may represent the potential initial topaz’s crystallization temperature from supercritical fluids in a pegmatite system. The log(fH_2_O/fHF^)fluid^ (1.27 (0.06)) is coherent with the fluorine activity calculated for hydrothermal fluids (pegmatitic stage) in equilibrium with the forming mineral (log(fH_2_O/fHF)^fluid^ = 1.2–6.5) and clearly different from pure magmatic (granitic) residual melts [log(fH_2_O/fHF)^fluid^ < 1]. The modelled H_2_O saturated fluids with the F content not exceeding 1 wt% may represent an anomalous water-dominant / fluorine-poor pegmatite lens of the Padre Paraíso Pegmatite Field.

## Introduction

Topaz is one of the principal fluorine-bearing silicates that occurs as an accessory mineral in fluorine-rich silicate rocks (rhyolites and granites) associated with pneumatolytic/hydrothermal events, and in ultrahigh-pressure rocks^1,2^. Its composition ranges from a nearly OH-free end member, Al_2_SiO_4_F_2_, in acid igneous rocks, to Al_2_SiO_4_F_1.4_(OH)_0.6_, with X_OH_ = OH/(OH + F) = 0.30, in hydrothermal deposits^3^. Higher OH content was reported for topaz found in ultrahigh-pressure (UHP)-rich Topaz–Kyanite quartzites from Hushan (west of Dongai), (X_OH_ = 0.35), and southern Sulu (X_OH_ = 0.40–0.55), eastern China^4^. A series of hydroxyl-rich topaz (OH-topaz) from X_OH_ = 0.22 up to the pure end-member Al_2_SiO_4_(OH)_2_, were synthesized^5^ at high-pressure/high temperature conditions (pressure from 5.5 to 10 GPa, temperature up to 1000 °C) in the Al_2_O_3_–SiO_2_–H_2_O system. For this reason, the study of the OH/F ratio plays a key role to understand the topaz’s formation ambient.

Usually, natural topaz with OH/(OH + F) < 0.5, crystallizes in the space group *Pbnm*, with one independent H-site, whereas in synthetic Al_2_SiO_4_(OH)_2 it_ displays two non-equivalent H-sites. The existence of an upper limit to the OH content is rationalized in terms of hydrogen located in two partially occupied sites whose occupancy limits the OH substitution for F to 50% (“proton-proton avoidance rule”)^6^. Conflicting results about the exact space group were reported for the synthetic end-member “topaz-OH”^7–9^. Northrup et al.^7^ found that in topaz-OH hydrogen atoms are spread over two independent crystallographic sites; one of these is not found in naturally occurring fluorine-bearing topaz. Both sites lie close to the mirror plane in the space group *Pbnm,* but their short distance (1.7 Å) prevents their simultaneous occupancy.

The proton environment in the topaz-OH was recently depicted by^10^ by first principle simulations on periodic systems, using the Hartree–Fock and Kohn–Sham self-consistent field method. As a result, two space groups, were reported: higher energy orthorhombic form *Pbnm* and a lower energy monoclinic form *P2*_*1*_*/c.*

The structural and elastic properties of topaz are strongly related to the fluorine content which determines the compressional behaviour of topaz; this causes polyhedral tilting and contraction as well as hydrogen bonding^10–12^. After ex situ heating at 950 °C for 18 h, the symmetry of topaz deviates from orthorhombic, suggesting *P1* as a possible space group^6^. The structural data were not reported in the paper, only the H-site coordinates (derived from the difference Fourier map) in the *Pbnm* and *P1* space groups were given. The response to increasing pressure and temperature of natural topaz from Gilgit division, Pakistan, was described by structure refinements from single crystal X-ray diffraction data^13^. Consequently, Al-O bond distances and the OH/F ratio controlling the thermal expansion and compression of topaz show an inverse relationship. IR and Raman spectroscopy^14–17^ carried out on both natural and synthetic topaz-OH explored the relationship between the structure and the strength of hydrogen bonds and suggested that additional phenomena like synergetic, cooperative, anti-cooperative or competitive effects govern the donor’s strengths and the acceptor’s capability. Two stretching OH bands (ν(OH)) due to the local F/OH ordering in opposite sites of the structure were distinguishable. Upon compression, the ν(OH) shifted to a lower frequency in topaz-OH and to a higher frequency in F-rich ones^16^. With increasing temperature, two phase transitions were detected: the first from *P1* to *Pbn2*_*1*_ (~ 135 °C), involving only the H atoms; the second (~ 160 °C), from *Pbn2*_*1*_ to *Pbnm* due to local F/OH ordering in the crystal structure^18^. Consequently, the real concentration and distribution of F and OH in natural topaz crystals can help to understand how the hydrogen bond geometry influences the OH behaviour with temperature, but not with any certainty. Understanding these mechanisms is important in order to determine the hydrophilicity and the exchange reactions of fluorine by hydroxyl groups, and ultimately to characterize the environmental redox conditions (H_2_O/F) required for the formation of topaz.

In spite of a large number of studies, as briefly reviewed above, no detailed thermal structural investigations on natural topaz have been conducted so far, preventing a rigorous modelling of the OH/F substitution in these minerals. Therefore, uncertainty about the H_2_O/F partitioning with the environment remains.

The aim of this work is therefore to investigate in depth the OH/F substitution in topaz in order to highlight the relationships between major element composition, structural features and geological environments. To this end, natural topaz from Minas Gerais (Brazil) were initially characterized by conventional chemical analyses by energy dispersive X-ray spectroscopy (EDS). These data provided important preliminary information about the composition of this group of topaz.

However, due to the light nature of the major elements forming this mineral (Si, Al, OH and F), which notoriously affect the analysis with EDS^19^, the chemical characteristics of this mineral need a more accurate determination in order to propose a robust chemical-physical model for crystal structure and crystallization conditions. Two selected crystals were subsequently investigated using a combination of unconventional X-Ray sources and neutron powder diffraction.

In situ synchrotron X-ray powder diffraction (XRPD) was selected to obtain powder patterns of higher accuracy and precision, because of the high intensity, angular resolution and beam collimation being considerably higher than those of conventional laboratory diffractometers. Additionally, horizontal polarization and tuneable X-ray wavelength reduced the fall in intensity as a function of 2*θ* as well as the high background level due to the sample fluorescence.

Compared to X-ray powder diffraction, neutron powder diffraction is the best technique to detect light atoms (e.g., H, C and N), as well as discriminate between those with a similar Z atomic number because the scattering powder is independent of the atomic number, the momentum transfer Q and the θ angle. The complementarity of X-ray and neutron data combined with the high flexibility of the experimental set-up of non-conventional radiation offers several advantages, such as the possibility to fully understand the nature of bonding and monitor in real-time the different processes taking place at the atomic level upon heating, even when light elements (i.e. H) or neighbouring elements (Si/Al and O/F) of the periodic table are present^20–22^.

Temperature-dependent variation of the unit cell parameters and transient structural modifications upon heating were highlighted in the 298–1273 K temperature range combining synchrotron X-ray and neutron diffraction data.

To complete the information, the Rietveld refinement of powder diffraction data collected on samples heated in situ at selected temperatures was performed in order to observe: (a) the real symmetry of the selected topaz and its proton environment; (b) the concentration and distribution of F and OH in the selected sample; (c) the correlation as a function of temperature between thermal expansion, fluorine content and the unit cell parameters; (d) the T-(P) conditions for topaz formation in the natural environment.

### Sample description and geological setting

**Figure 1:**
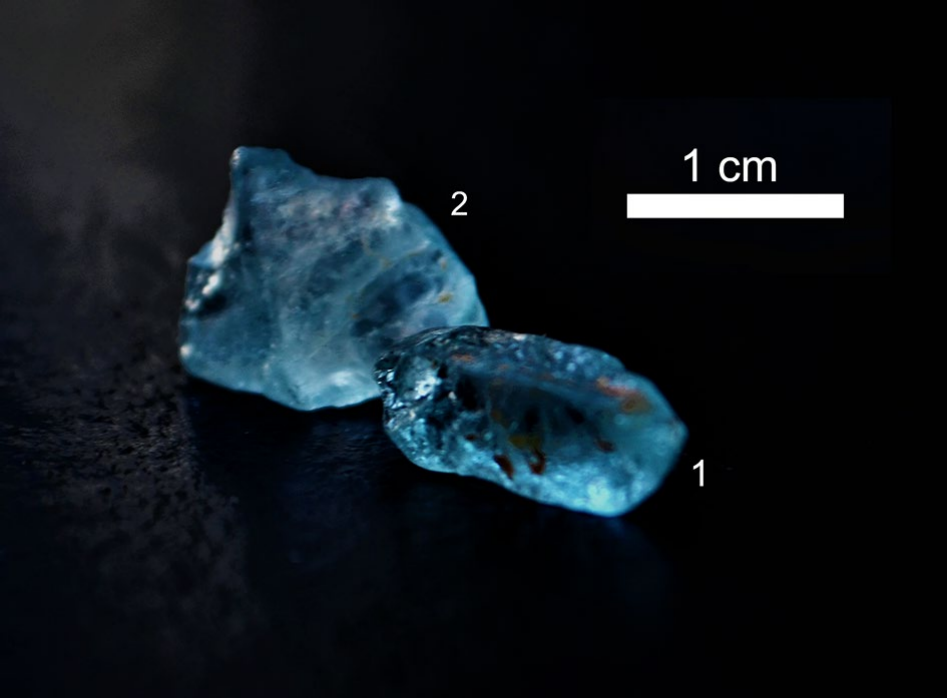
Topaz from Padre Paraíso municipality in Minas Gerais State in Brazil. Dimensions are 1 × 0.5 × 0.3 cm and 1 × 1.3 × 0.4 cm for crystal 1 and 2, respectively. They both are light blue in colour with good transparency and of medium–high clarity. Impurities (brownish-red area) were removed before chemical and structural analyses. The pic has been cropped with GIMP-GNU image manipulation program (https://www.gimp.org/) and the scale has been added with a Microsoft Office tool.

Coloured topaz from Padre Paraíso (North-East of Minas Gerais State in Brazil) (Fig. 1), from now on called PadPar, was studied. This locality is renowned for the production of gemstones associated with granitic magmatism and pegmatitic events of the Eastern Brazilian Pegmatite Province^23,24^.

The pegmatite body is related to granitic residual melts crystallized between 630 and 480 Ma (zircon U–Pb dating^24^). Pegmatite events followed the “G5 granites and charnokites” unit of the Eastern Brazilian Pegmatite Province. The G5 supersuite is a magmatic event of post-collisional origin related to the gravitational collapse of the Araçuaí orogen^24^; zircon and monazite U–Pb ages and zircon Pb-Pb ages constrain the evolution of the G5 supersuite from 520 to 480 Ma^24–26^. The G5 unit is a porphyritic biotite granitoid, classified as a Type-I granite that is metaluminous and K-rich^24^. Associated to the “G5 granite” is the Padre Paraíso charnockite^24^, which is a porphyritic biotite rich granitoid that has a greenish colour. Its primary mineral assemblage is composed of K-feldspar, plagioclase, biotite, hornblende and hypersthene^23^. Our topaz present a clear to intense light blue colouration, with no fades or colour flaws in all the samples collected. All crystals have good transparency with medium–high clarity. They are poorly to very poorly included, some solid inclusions of quartz and micas are recognizable. Some liquid or biphasic inclusions are also present probably due to their pegmatitic origin. All the liquid inclusions are iso-orientated. The refractive index is high, ranging from 1.620–1.631 to 1.634–1.642. For this work, only samples without inclusions were selected.

## Methods

### Scan electron microscopy and EDS microanalysis

For this study a CAMSCAN MX 2500 Scan Electron Microscope (SEM) with an EDAX system for Energy Dispersive Analyses were used. The SEM was equipped with a high brightness LaB6 cathode, operating in a high vacuum. The samples were coated with carbon. For quantitative purposes, a set of natural minerals was used as standards for their specific elements: augite (Si and Ca), fluorite (F), gahnite (Al) and olivine (Mg, Fe). Relative analytical errors (1σ) of major elements were below 1.00% for Si and for Al; and 3.00–6.00% for F.

Images were collected both in SEI (secondary electron imaging) and BEI (backscattered electron imaging). The parameters used for the analyses were EHT 20 kV, EMI 71µA, FIL 1.80A with a working distance of 25 mm. Imaging and measurements were performed at the Microscopy Laboratory of the University of Padua.

### X-ray and neutron powder diffraction measurements and structure determination

The PadPar powdered samples were poured into a thin-walled boron capillary (diameter 0.5 mm) and then collected in transmission geometry (monochromatic wavelength of 0.827 Å (15 keV) and 1 × 0.3 mm^2^ spot size at the MCX beamline^22,27^ of Elettra—Synchrotron Trieste, (Italy). They were then mounted on a standard goniometric head, and spun during data collection. Powder diffraction patterns were collected in 10°–65° 2θ range with a step size of 0.008° and an exposure time of 1 s.

Each sample was subjected to the same heat treatment; samples were heated from room temperature to 1273 K with a heating rate of 5 K/min using a hot gas blower directing a hot air flux onto the spinning quartz-capillary. Diffraction data were collected every 50 K. The temperature was continuously measured by a thermocouple, and calibrated using the quartz thermal expansion and phase transition.

Neutron Powder Diffraction (NPD) experiments were then carried out on the high-flux two-axis neutron powder diffractometer D20 at the Institut Laue Langevin (ILL, Grenoble, France) using the same topaz sample. Useful data were collected between 6° and 142°, of which 22° to 142° were treated as follow. The samples were heated in situ under flowing gas (5% H_2_/He), from room temperature to 1273 K (heating rate 2 K/min). Data sets were collected for 2 min, thus covering 4 K. A wavelength of 1.54 Å was chosen, from a germanium-(115) monochromator at 90° take-off angle. The sample was poured into a 4 mm diameter vanadium cylinder, placed in the centre of the furnace’s vacuum vessel and heated by a 30 mm diameter vanadium resistor^28^. A type-K thermocouple was located in the centre of the furnace in order to calibrate the temperature. The same configuration was maintained during all data collection.

A set of diffraction patterns were obtained with this procedure as a function of temperature. All data processing was carried out by the full profile Rietveld analysis using the GSAS package^29^ with the EXPGUI interface^30^. The profile was modelled by a pseudo-Voigt function which uses an accurate description of the reflection asymmetry due to axial divergence described by^31^ as an implementation of the peak shape function described by^32^. The background was empirically fitted using a Chebyschev polynomial with 20 and 14 polynomial coefficients for synchrotron and neutron refinements, respectively. The scale factor, 2θ-zero shift, unit-cell parameters and thermal displacement parameters were accurately refined. Final crystal and refinement data for four selected temperatures (298, 776, 1073 and 1273 K) are reported in Tables 1 and 2. The thermal expansion coefficients were investigated for both samples from 298 to 1273 K (Table 3) by the EosFit7-GUI software ^33^. Positional and thermal parameters, fractions at four selected temperatures (298, 776, 1073 and 1273 K) can be found in Supplementary Tables S1 and S2, for synchrotron and neutron refinements, respectively.

**Table 1:** Refinement details of the synchrotron data collection and unit cell parameters of topaz at 298, 776, 1073 and 1273 K.

	298 K	776 K	1073 K	1273 K
Space group	*Pbnm*	*Pbnm*	*Pbnm*	*Pbnm*
*a* (Å)	4.65237 (2)	4.66991 (2)	4.68036 (2)	4.68413 (1)
*b* (Å)	8.80479 (3)	8.83312 (4)	8.85117 (4)	8.85763 (1)
*c* (Å)	8.38936 (3)	8.42725 (1)	8.44936 (4)	8.45630 (1)
*V* (Å^3^)	343.655 (2)	347.623 (3)	350.029 (3)	350.851 (6)
2*θ* range (°)	10–55	10–55	10–55	10–55
*R*_wp_ (%)	7.81	7.49	8.4	9.26
*R*_p_ (%)	5.89	5.62	6.25	6.95
*R*_F_^2^ (%)	5.16	4.75	7.31	9.35
No. of reflections	5631	5631	5631	5631
*N*_obs_	184	184	186	186
*N*_Var_	98	98	98	98

R_p_ = Σ[Y_io_ − Y_ic_]/ΣY_io_; R_wp_ = [Σwi(Y_io_ − Y_ic_)^2^/ΣwiY_io_^2^]^0.5^; R_F2_ = Σ|F_o_^2^ − F_c_^2^|/|F_o_^2^|.

**Table 2:** Refinement details of the neutron data collection and unit cell parameters of topaz at 298, 776, 1073 and 1273 K.

	298 K	776 K	1073 K	1273 K
Space Group	*Pbnm*	*Pbnm*	*Pbnm*	*Pbnm*
*a* (Å)	4.6445 (1)	4.6597 (1)	4.6703 (3)	4.6845 (8)
*b* (Å)	8.7877 (6)	8.8132 (5)	8.8298 (5)	8.8518 (16)
*c* (Å)	8.3742 (6)	8.4066 (5)	8.4286 (6)	8.4610 (17)
*V* (Å^3^)	341.79 (4)	345.23 (4)	347.58 (4)	350.85 (11)
2*θ* range (°)	22–142	22–142	22–142	22–142
*R*_wp_ (%)	8.95	8.55	7.80	6.45
*R*_p_ (%)	6.47	6.08	5.67	5.04
*R*_F_^2^ (%)	5.03	7.69	10.26	11.52
No. of reflections	2409	2406	2406	2366
*N*_obs_	412	377	377	655
*N*_Var_	98	98	98	98

R_p_ = Σ [Y_io_ − Y_ic_]/ΣY_io_; R_wp_ = [Σwi(Y_io_ − Y_ic_)^2^/ΣwiY_io_^2^]^0.5^; R_F2_ = Σ|F_o_^2^ − F_c_^2^|/|F_o_^2^|.

**Table 3:** Thermal expansion coefficient for all crystallographic values calculated from synchrotron and neutron diffraction data, respectively.

	*a*	*b*	*c*	*V*
298-1300 K Synchrotron data	a_0_ = 4.6524 (1)	b_0_ = 8.8048 (1)	c_0_ = 8.3894 (1)	V_0_ = 343.66 (1)
	α_0_ = 0.64 (2)	α_0_ = 0.51 (2)	α_0_ = 0.83 (3)	α_0_ = 1.96 (6)
	α_1_ = 0.12 (3)	α_1_ = 0.19 (2)	α_1_ = 0.04 (5)	α_1_ = 0.36 (9)
	α_2_ = 0.00000	α_2_ = 0.00001	α_2_ = 0.00000	α_2_ = 0.00002
298-1300 K Neutron data	a_0_ = 4.6441 (6)	b_0_ = 8.7879 (1)	c_0_ = 8.3738 (13)	V_0_ = 341.77 (14)
	α_0_ = 0.57 (11)	α_0_ = 0.45 (10)	α_0_ = 0.60 (12)	α_0_ = 1.50 (30)
	α_1_ = 0.26 (15)	α_1_ = 0.29 (12)	α_1_ = 0.42 (16)	α_1_ = 1.20 (40)
	α_2_ = 0.00010	α_2_ = 0.00002	α_2_ = 0.00001	α_2_ = 0.00008

Thermal expansion coefficients were calculated by EosFit7-GUI software^31^.

## Results

### Mineral chemistry

The two Padre Paraíso topaz samples show compositional homogeneity, with a very low fluorine content (F = 9.95–10.00 wt%), SiO_2_ = 33.18–33.58 wt% and Al_2_O_3_ = 56.42–56.87 wt%. The OH/(OH + F) ratio ranges from 0.474 to 0.476. Recalculation on the basis of three cations, indicate an almost ideal Si and Al occupancy, which suggests that the hydroxyl site possibly contain small amount of waters to balance the scarceness of F in the site. Detailed chemical analyses are provided as Supplementary Tables S3.

### Crystal structure models at room temperature

**Figure 2:**
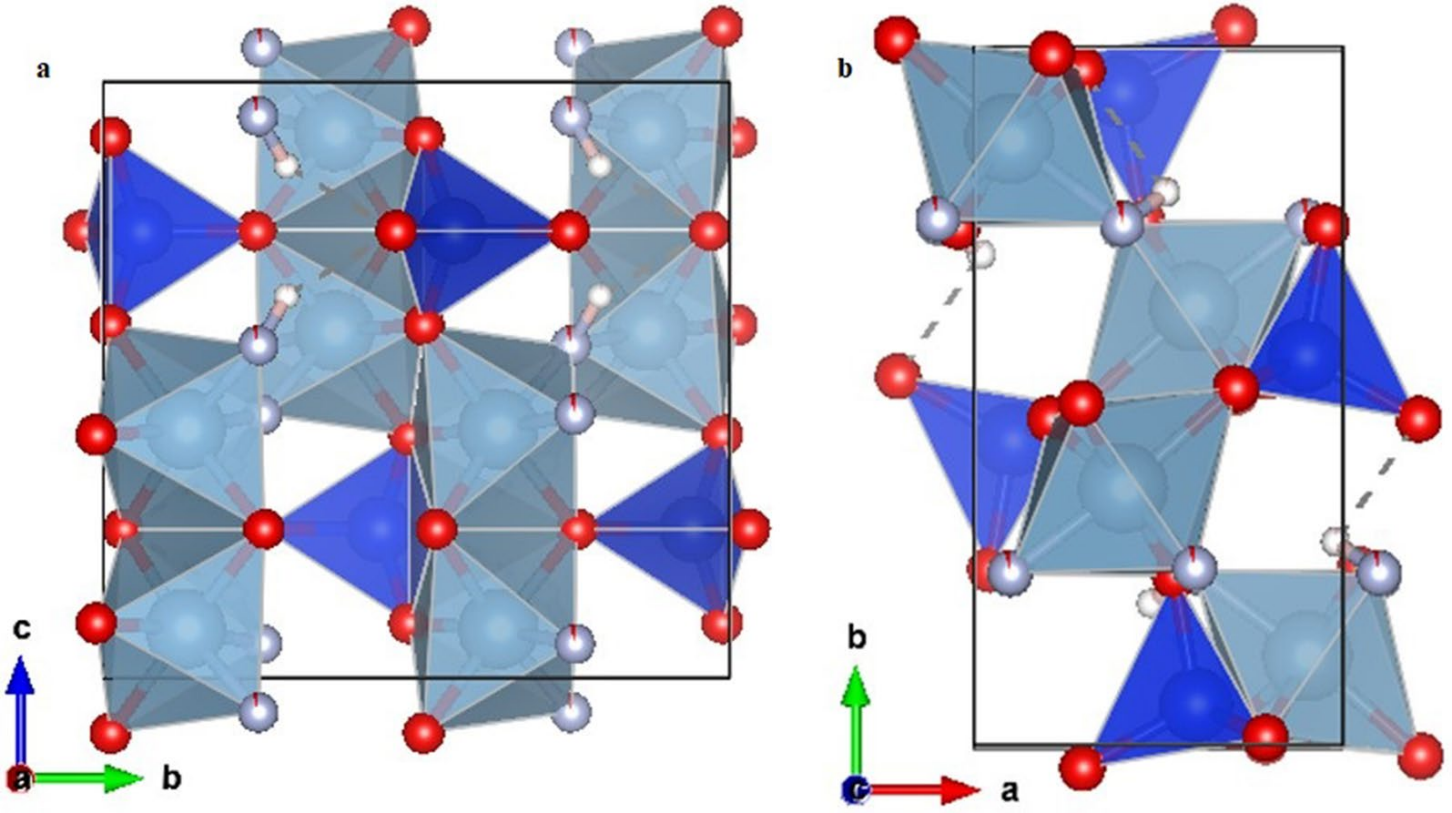
Projection of the topaz structure along the *a* (**a**) and *c* axes (**b**), respectively. The tetrahedral [SiO_4_]^4−^ groups, in blue, are linked to octahedral chains of Al[O_4_(F,OH)_2_], in grey, in a zig zag fashion parallel to the *c*-axis. The smaller red, grey and white atoms are O, F and H, respectively. The structure image has been obtained using VESTA Version 3 (https://jp-minerals.org/vesta/en/download.html).

The topaz structure consists of [SiO_4_]^4−^ groups linking octahedral chains of Al[O_4_(F, OH)_2_] in a zig-zag arrangement parallel to the *c*-axis. Four out of the six anions surrounding the Al^3+^ ion belong to [SiO_4_]^4−^tetrahedra; and two form F − or OH − groups (Fig. 2a,b). Natural topaz crystallizes in the orthorhombic *Pbnm* space group; pronounced sectoral textures with growth planes *{hkl}* optically triclinic, *{0kl}, {k0l},* and *{hk0}* optically monoclinic, and *{100}, {010}*, and *{001}* optically orthorhombic are also well documented^34,35^.

As expected, synchrotron X-ray powder diffraction of PadPar topaz yields higher quality powder diffraction patterns than when compared to diffraction, hence this data set was chosen for the initial structural investigation. The indexing process performed by EXPO2014 via the N-TREOR09 program^36^ unambiguously suggested the *Pbnm* space group. Consequently, PadPar structural refinements of data collected at room temperature were carried out in the space group *Pbnm* starting from the atomic coordinates reported by^11^ without the proton position and using only the (neutral) atomic scattering factors of Al, Si, and O. When the convergence was achieved, no peaks larger than ± 0.32 e^−^/Å^3^ were present in the final difference Fourier map. Lattice parameters refined from synchrotron X-ray are: *a* = 4.652373(18) Å, *b* = 8.804789(34) Å, *c* = 8.389362(32) Å, *V* = 343.6548(23) Å^3^.

The structural refinement with the neutron diffraction data collected at room temperature was performed in the space group *Pbnm*, starting with the atomic coordinates obtained from the X-ray structural refinement.

The F-amount of our sample refined on the basis of the neutron diffraction data is 1.032 a.f.u; this correspond to 10.94 wt% (Table 4), in agreement with the EDS microanalysis. When convergence was achieved, the final difference Fourier map revealed the occurrence of a peak at *x* = *0.020, y* = *0.707, z* = *0.141* which was then refined with the H scattering length. The O–H bond distance was initially fixed and the constrain was completely removed in the last cycles of refinement (O4-H 0.979 (4) difference < 2σ) whereas the proton occupancy factor was fixed as a function of the oxygen at the F/O4 site (for the F/O4 site, %O = 100 − %F).

**Table 4:** The refined neutron bond distances between the proton site and the surrounding anions.

	This work	^11^
F/O4···F/O4 (Å)	3.262 (7)	3.195 (3)
H···O1 (Å)	2.204 (9)	2.307 (6)
H···O2 (Å)	2.186 (7)	2.216 (5)
H···O3 (Å)	2.432 (7)	2.380 (5)
H···F/O4 (Å)	0.979 (7)	0.989 (5)
H···H (Å)	1.715 (7)	1.463 (5)

The corresponding values refined by^11^ are reported for comparison.

**Figure 3:**
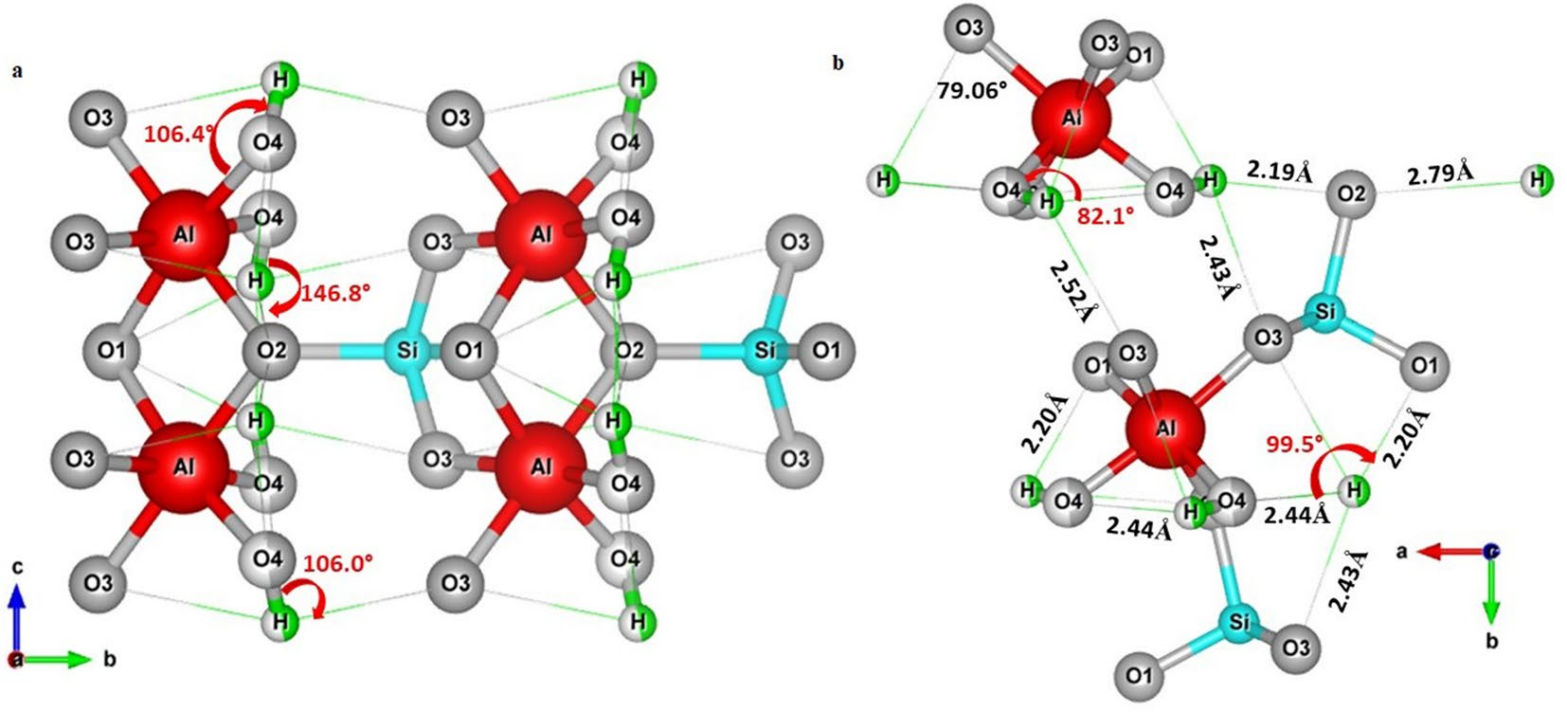
Hydrogen-bond system of topaz. Schematic representation of hydrogen bonding (green lines) at room temperature. Colours as in Fig. 2. The main interactions between H atoms and the surrounding polyhedral environment are viewed along the *a* (**a**) and *c* axes (**b**). The bond distance and angle between the proton site and the surrounding anions revealed the occurrence of potential H···O/F interactions, where two of them (H···O1 and H···O2) are slightly stronger than the others. The H sphere appears partially green coloured due to the OH/F substitution, according to the refined occupancies from neutron diffraction data. The structure image has been obtained using VESTA Version 3 (https://jp-minerals.org/vesta/en/download.html)^76^.

The unit cell parameters obtained from the neutron data are: a = 4.64453(3) Å, b = 8.7877(6) Å, c = 8.3742(6) Å and V = 341.79 Å3, in very good agreement with those obtained from synchrotron diffraction data. The H and F occupancies refined from neutron diffraction were then fixed in the synchrotron structure refinement. The bond distances and angles between the proton site and the surrounding anions revealed the occurrence of potential H···O/F interactions (Fig. 3a,b). Two of them (H···O1 and H···O2) are slightly stronger than the others (Table 4). This OH topological configuration reveals shorter H···O/F bond distances and longer H–H bond distances longer (H–H 1.715(6) Å) than those previously described (1.463(5) Å^11^) (Fig. 3a,b; Table 4).

### Temperature-dependent variation of the unit cell parameters and structural modifications

**Figure 4:**
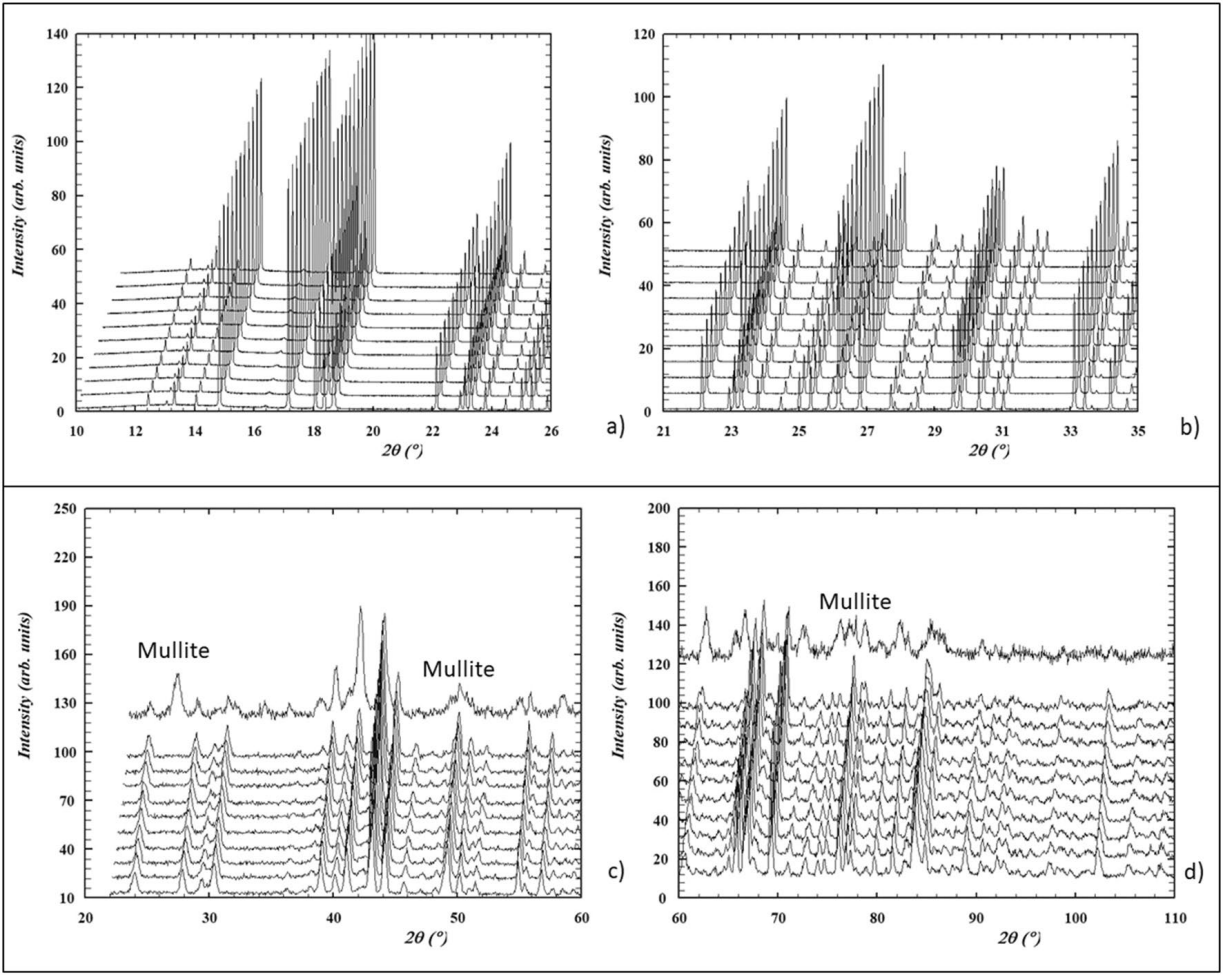
Cascade plots of PadPar topaz from Synchrotron (**a**, **b**) and Neutron (**c**, **d**) diffraction data. The samples were heated from room temperature to 1273 K (heating rate 5 K and 2 K/min for Synchrotron and Neutron data collection, respectively). Powder diffraction patterns recorded every 100 K are also shown. In neutron analyses, the rapid decomposition of the sample upon heating revealed the formation of mullite Al_4+2x_Si_2−2x_O_10−x_. This image has been obtained with WinPLOTR from FullProf Suite (https://www.ill.eu/sites/fullprof/index.html)^77^.

**Figure 5:**
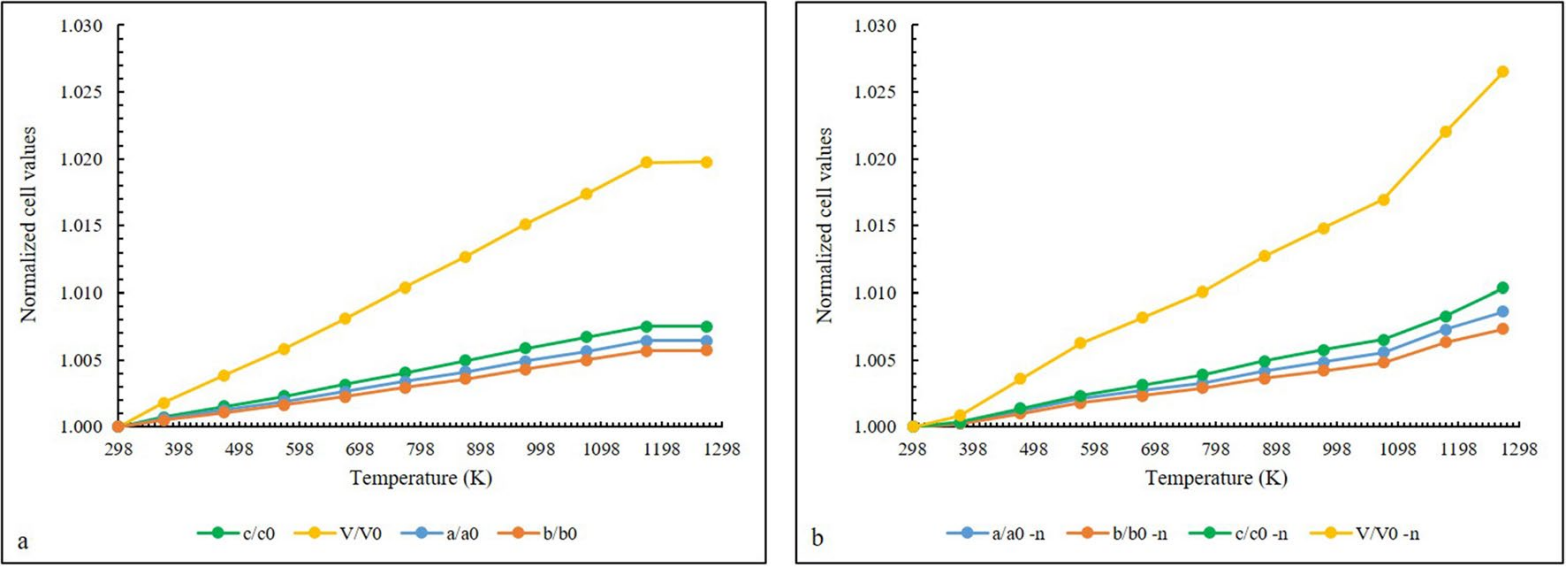
Evolution of unit cell parameters normalized with respect to room temperature values (*a/a*_*0*_, *b/b*_*0*_, *c/c*_*0*_, *V/V*_*0*_) from in situ synchrotron (**a**) and neutron diffraction (**b**) data. A strong change in the unit cell parameters evolution is detected from neutron data (**b**), in particular, up to 1075 K *a* and *b* cell-axes have a similar expansion rate while the *c*-axis undergoes an increase up to about 1273 K. Standard deviation errors are within the symbol size.

The XRPD temperature ramp (Fig. 4a,b) analysis revealed that topaz maintains its crystallinity and symmetry up to the highest investigated temperature (T = 1273 K). Instead in neutron data (Fig. 4c,d), a progressive broadening peak was observed from ~ 1170 K, thus highlighting a progressive loss of crystallinity. This result can be explained by the different heating rate used during the data collection, as discussed below. The evolution of the cell parameters of the PadPar samples during the in situ heating process in the 298–1273 K range from both synchrotron and neutron data are reported in Fig. 5a and b, respectively. To better understand these differences and to allow a better comparison between the two systems and among their whole cell parameters, we reported normalized dimensionless values defined as *V(T)/V*_*0*_*, a(T)/a*_*0*_*, b(T)/b*_*0*_, and *c(T)/c*_*0*_, being the reference values obtained in the refinement of the first recorded pattern (T) 298 K.

In both samples *a*, *b* and *c* increase as the temperature increases up to 1010 K, indicating that the thermal expansion is the physical mechanism dominating this stage of the experiment. Unit-cell axes refined from synchrotron data do not show any other modifications until the maximum temperature is reached. A strong change in the unit cell parameters evolution is detected from neutron data. In particular, up to this temperature *a* and *b* cell-axes have a similar expansion rate while the *c*-axis undergoes a significant increase up to about 1273 K. These variations are reflected in the evolution of the unit-cell volume, *V*_*S*_ (δ*V* = 2%) and *V*_*N*_ (δ*V* = 2.65%).

The thermal expansion coefficients were investigated for both samples from 298 to 1273 K using the EosFit7-GUI software^33^. The temperature evolution was properly described using a polynomial expression:

1 $$\alpha = \alpha_{0} + \alpha_{1} T + \alpha_{2} T^{ - 1}$$

where the mean thermal expansion coefficient α is expressed in K^–1^, and constants α_0_, α_1_, and α_2_, derived from the experimental data are expressed in K^–1^, K^–2^, and K, respectively^37^.

**Figure 6:**
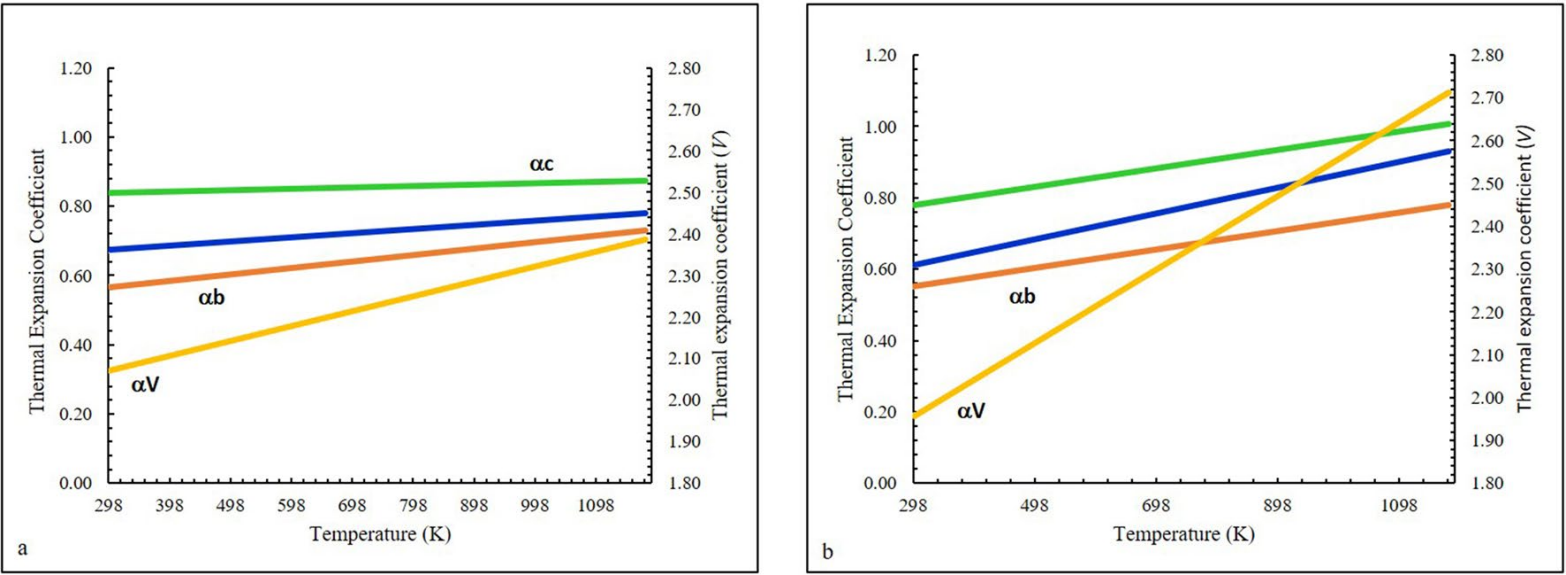
Thermal expansion model for in situ heating of the PadPar topaz, obtained from synchrotron (**a**) and neutron diffraction (**b**) analyses in the temperature range of 298 to 1273 K. Orange, yellow, green and blue symbols refer to Temperature *vs* Volume data selected for the EoS fitting (see text for details). Standard deviation errors are within the symbol size.

**Figure 7:**
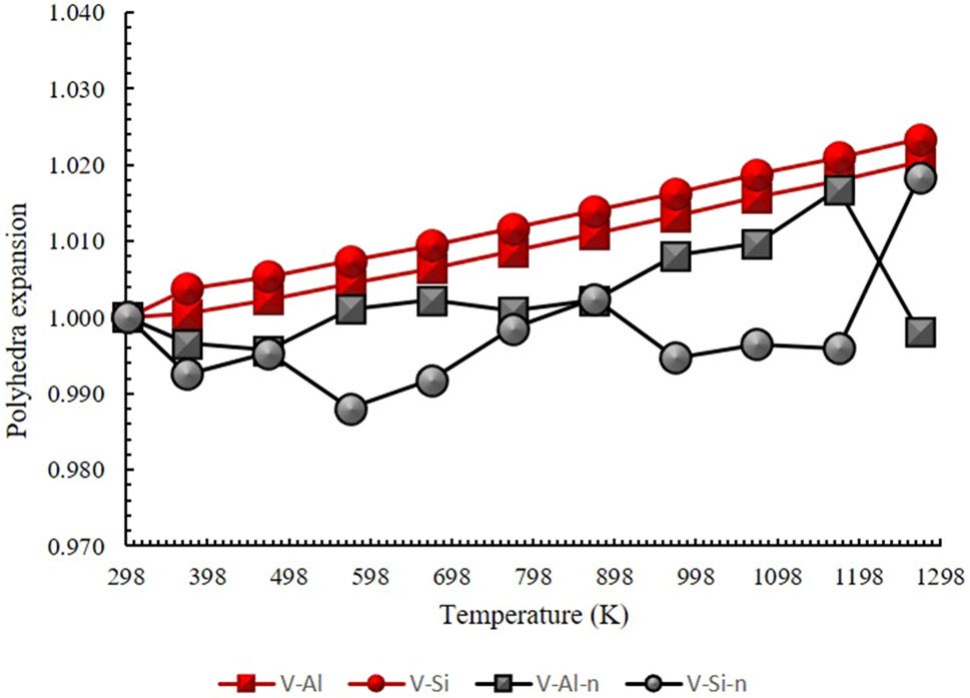
Polyhedral evolution of topaz expressed as *V*/*V*_0,_ with V–Al and V–Si indicating octahedral and tetrahedral volume respectively, as obtained from synchrotron (red symbols) and neutron (grey symbols) data. For in situ neutron data the octahedral expansion is regularly counterbalanced by a tetrahedral contraction, up to 1170 K. Above this temperature, the fluorine loss induced a structural modification that suddenly change this trend. More details are reported in Fig. 8. Standard deviation errors are within the symbol size.

The PadPar coefficients along the crystal axes are α_*a*_ = 6.40(20) × 10^–6^ K^−1^, α_*b*_ = 5.09(17) × 10^–6^ K^−1^, α_*c*_ = 8.30(30) × 10^–6^ K^−1^, α_*V*_ = 1.96(6) × 10^–5^ K^−1^, then the ratio of thermal expansion coefficients *α*_*a*_:*α*_*b*_:*α*_*c*_ is *0.77*:*0.61*:*1*. These values are in very good agreement with those reported by Tennakoon et. al.^38^. For neutron data the coefficients are α_*a*_ = 5.70(11) × 10^–6^ K^−1^, α_*b*_ = 4.50(10) × 10^–6^ K^−1^, α_*c*_ = 6.00(12) × 10^–6^ K^−1^, α_*V*_ = 1.50(3) × 10^–5^ K^−1^, then the ratio of thermal expansion coefficients *α*_*a*_*:α*_*b*_*:α*_*c*_ is *0.95:0.75:1*. These coefficients have been compared in Fig. 6a,b. PadPar coefficients are in-line-with those reported by^39^ showing a significant anisotropy due to the presence of a strong cleavage along {001}^40^. These changes are reflected on [SiO_4_]^4−^ tetrahedra and Al[O_4_(F,OH)_2_] octahedra. In fact, Si–O and Al-O distances and angles refined from synchrotron data increase linearly until the maximum temperature is reached, resulting in a positive expansion of the polyhedra (Fig. 7).

**Figure 8:**
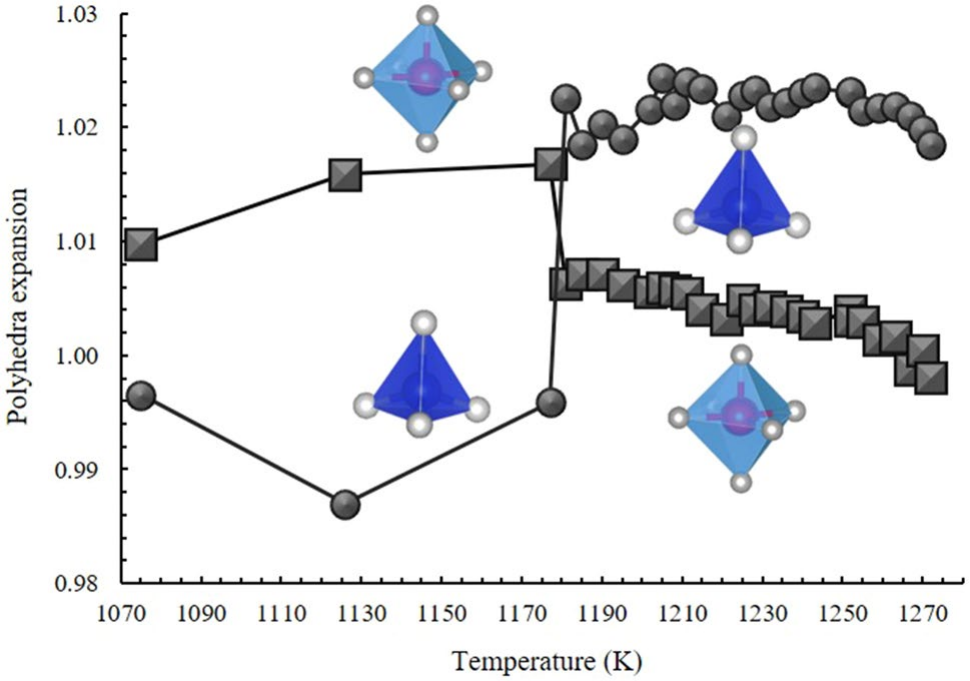
Polyhedral evolution of topaz in the critical zone (1071–1273 K) expressed as *V*/*V*_0_ as obtained from neutron data. The octahedral expansion (square symbols) is continuously balanced by a tetrahedral contraction (circle symbol) up to 1170 K, where a strong F loss, accompanied by a crystallinity loss and a phase change are envisaged. At higher temperature the concomitant expansion and contraction of tetrahedra and octahedra, respectively, is minimized. For sake of clarity octahedra (light blue) and tetrahedra (dark blue) are also represented.

A very different situation was encountered for in situ neutron data where the octahedral expansion is regularly counterbalanced by a tetrahedral contraction, up to 1170 K. Above this temperature, this trend suddenly changes as a result of the structural modifications induced by the fluorine loss, in close agreement with the evolution of refined occupancy fractions reported in Fig. 8.

**Figure 9:**
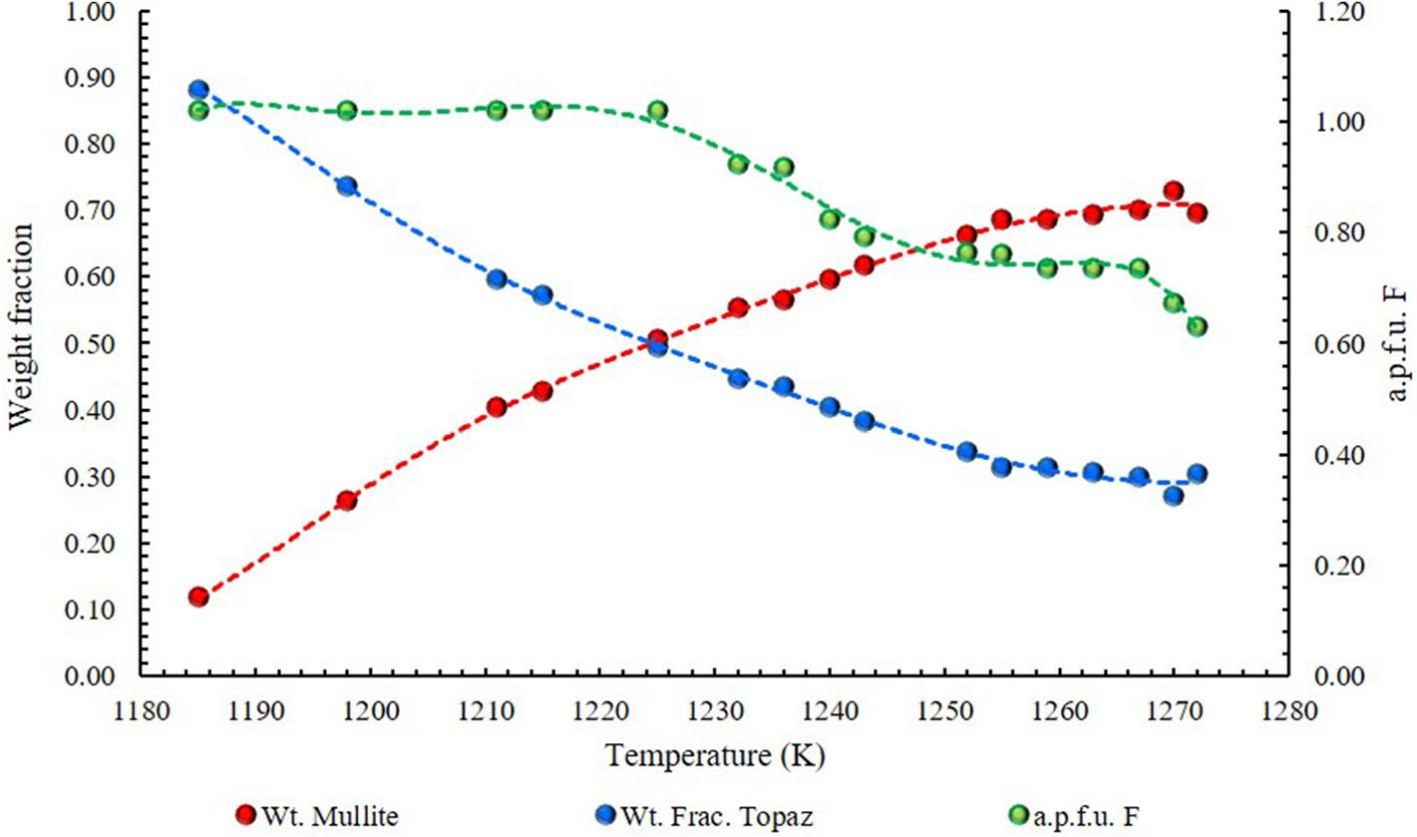
Fluorine behaviour in topaz-mullite transition zone (1170–1273 K; see also Fig. 5b). F atoms in topaz (atomic formula unit, a.p.f.u.: green pattern); weight fractions (wt. fract.) of topaz and mullite in blue and red patterns, respectively. At 1181 K topaz decomposition starts together with the appearance of a mullite phase (up to 0.10 wt. fract). F remains strongly partitioned in topaz up to 1225 K, but reduces the weight fraction of this phase in the system by 50%. At higher temperatures, in a very narrow increasing interval, fluorine is largely released by the topaz structure, reaching 0.80 a.p.f.u. at 1252 K and concurrently mullite becomes the dominant phase (~ 70%). This F content is stabilized in the remnant topaz (~ 30%) up to 1270 K.

In neutron analyses, the main diffraction peaks associated with the topaz phase declined rapidly with continued heating, indicating a rapid decomposition of the sample. A second phase appeared to grow at the same rate as the peak from the previous phase declined thus revealing the formation of mullite Al_4+2x_Si_2-2x_O_10-x_ ((120) and (210) reflections, at 25.90 and 26.09 2θ°, respectively). Mullite occurrence is well known in literature^40,41^, but always at higher temperature. When the highest temperature was reached, the Rietveld refinement indicated ~ 30 and 70% in weight of topaz and mullite, respectively. At the same time, the fluorine content decreased from 0.77 to 0.63 a.f.u (Fig. 9).

## Discussion

Combined synchrotron and neutron diffraction data collected in this study allowed us for the first time with a rigorous analytical strategy, to infer, that the real symmetry of PadPar topaz is orthorhombic *Pbnm* (Tables 1 and 2). Hydrogen atoms are hosted in only one site in good agreement with those reported for a natural topaz, with differences < 2σ^11,42^. On the basis of the neutron diffraction data, the F-amount gives rise to ~ 1.03 a.p.f.u. so the chemical composition can be inferred as being Al_1.92_Si_0.96_O_4.00_F_1.032_OH_0.968;_ (OH/(OH + F) = 0.484). The fluorine content (10.94 wt%) appears to be in very good agreement with that measured by EDS, but extremely low with respect to the value obtained with the correlation equation (~ 18.5% wt) proposed by^2^. This last correlation is widely used to estimate the fluorine content in topaz, but our results, as well as those reported by^11^ reveal that the empirical correlation between F contents and lattice parameters is not always satisfactory.

Unit cell parameters increase as the temperature increases (Fig. 5b) up to 1010 K, indicating a positive thermal expansion that dominate this stage of the experiment. Above this temperature, both fluorine content and topaz crystallinity decrease, and mullite starts its growth over topaz. According to^41^ mullite nuclei may form randomly on the surface of topaz particles from the very beginning thus protecting it from further decomposition. This reaction is self-catalysed by SiF_4_, and its occurrence is strongly dependent on several factors such as air flow, heating rate and fluorine concentration.

The analytical strategy applied here, therefore, was successful in determining the fluorine content in topaz, and its behaviour with increasing temperature. The F/OH ratio in this phase is crucial not only for the forming gem process, but also to better understand the circulation of fluids (H_2_O/F) in the forming environment.

### Variation of log(***f***H_2_O/***f***HF)^fluid^ of the inferred fluid based on F–OH concentrations of topaz

The topaz forming system is H_2_O saturated peraluminous, melt or/and fluid(s) with low calcium and F contents > 1 wt%^43,44^. In the late and post-magmatic evolution of any intrusive events, the residual melts (volatile—saturated in composition) and fluids tend to escape to higher structural levels, or lose their identity due to the interaction with the already crystallized phases, or due to a continuous interaction with the host rock (i.e. ^45–47^). For the sake of clarity, in the following sections we use the equilibrium equations and formalism applied to the fluid state.

The OH-F substitution in the topaz solid solution was estimated to not exceed X_OH_ = 0.5 [X_OH_ = OH/(OH + F)] due to proton–proton avoidance^3^. However, OH-rich topaz with X_OH_ = 0.54 occurred in samples from ultrahigh-pressure rocks of the Sulu terrane, eastern China^2^, as well as in high pressure experimental products, indicating that depending on the P–T–X-conditions, topaz might be stable along the complete (OH,F)-solid solution series^5,48^. The effective ionic radius and electronegativity of F- and OH are very close^49,50^, therefore it is reasonable to assume an ideal site mixing of F and OH in various F-OH minerals, including topaz^50^.

Biotite is the most widely used mineral to estimate the halogen content of fluids in various magmatic-hydrothermal systems, according to the experimentally well calibrated exchange reaction^51^:

$$xBio\left( {OH} \right)_{2} + \, 2HX \, = \, xBio\left( X \right)_{2} + \, H_{2} O$$

where X = F and Cl and xBio = Mg cation numbers/sum octahedral cation numbers in biotite. Various empirical equations are thus proposed to estimate the halogens fugacity for the fluids in equilibrium with biotite^52,53^, and the application of this method facilitated the investigation of the F–Cl–OH partitioning between biotite and fluids in various magmatic-hydrothermal systems^53–61^. Following the same line of reasoning, we calculated the fluorine fugacity [expressed as the ratio log(*f*H_2_O/*f*HF)] of possible fluids (or H_2_O-F saturated) coexisting with PadPar topaz. The refinement of F/OH occupancy from neutron data, allowed the consolidation of the chemical data for the F contents in the PadPar topaz. This is an anomalous fluorine-poor topaz, with X_OH_ (0.456–0.476), close to the physical proton–proton avoidance. As mentioned above, the low to very low fluorine content (X_OH_ = 0.54) of natural topaz is observed in ultrahigh pressure metamorphic terrains^4^ but is rarely observed in the topaz of late or post magmatic origin from Minas Gerais fields^61^.

Notwithstanding, it is worth noting that among the worldwide late or post magmatic topaz^62–65^, those from the Padre Paraíso pegmatite are undoubtedly a fluorine poor type (PadPar: F ~ 10.0–10.94 wt% versus worldwide average F ~ 18 wt%) and, to the authors knowledge, this topaz type of the Minas Gerais pegmatites has never been investigated before. Therefore, it is intriguing to determine the fluid activity in the PadPar pegmatite body and the fluorine and OH topaz contents (as determined by the proposed analytical protocol) can be utilized as indicators of the F (and OH) contents of fluids in equilibrium with this gem.

It is high challenging to extrapolate the ambient of mineral formation from the crystal itself, since it is often doubtful that collected samples truly represent the in situ conditions at which minerals formed. However, due to the fairly constant major-element composition of this mineral species, the OH/F concentration ratio and fully characterized crystal structure (site occupancy) may reflect the nature of the fluid composition from which topaz formed.

The (*f*H_2_O/*f*HF)^fluid^ are calculated from the concentrations of F, and OH on the mixed site in topaz octahedra using the empirical equations proposed by^3,66^, relating the equilibrium constants of F-OH exchange in topaz:

$${\text{Al}}_{{2}} {\text{SiO}}_{{4}} \left( {{\text{OH}}} \right)_{{2}} + {\text{2HF }} = {\text{ Al}}_{{2}} {\text{SiO}}_{{4}} \left( {\text{F}} \right)_{{2}} + {\text{H}}_{{2}} {\text{O}}$$

The thermodynamic properties describing the partitioning of F-(Cl)-OH between minerals and late or post magmatic fluids are from^49,67^.

We do not know the final temperature of equilibration with the coexisting fluid(s) for the single crystals, so we calculated (*f*H_2_O/*f*HF)^fluid^ for a range of potential temperatures of equilibration in a 298–1273 K range. However, following the results obtained with synchrotron and neutron data and temperature-dependent structural modelling, which reveal that PadPar topaz maintain its crystallinity and symmetry up to 1170 K, we can argue that PadPar topaz started its nucleation at this temperature. This value is coherent with the topaz stability field in the system Na_2_O–Al_2_O_3_–SiO_2_–F_2_O^63^, but it is also a rather high temperature for the formation of granitic pegmatites, (1075–625 K^68–70^). However, liquidus temperatures of 1120 K was experimentally obtained for the initial crystallization of the topaz-albite granite assemblage from a supercritical fluid (~ 28 wt% H_2_O and ~ 45 wt% F completely miscible in all proportions at magmatic temperature and pressure)^71^.

According to a previous experimental work^3^, the pressure effects on (*f*H_2_O/*f*HF)^fluid^, at constant topaz composition (6.24/T in units of kbar^−1^) is negligible given the uncertainty in the thermodynamic data, therefore calculations were made at constant P = 1 kbar. The approach that we used is validated by several investigations on the fluorine activity in the magmatic-hydrothermal systems^53–58^.

**Figure 10:**
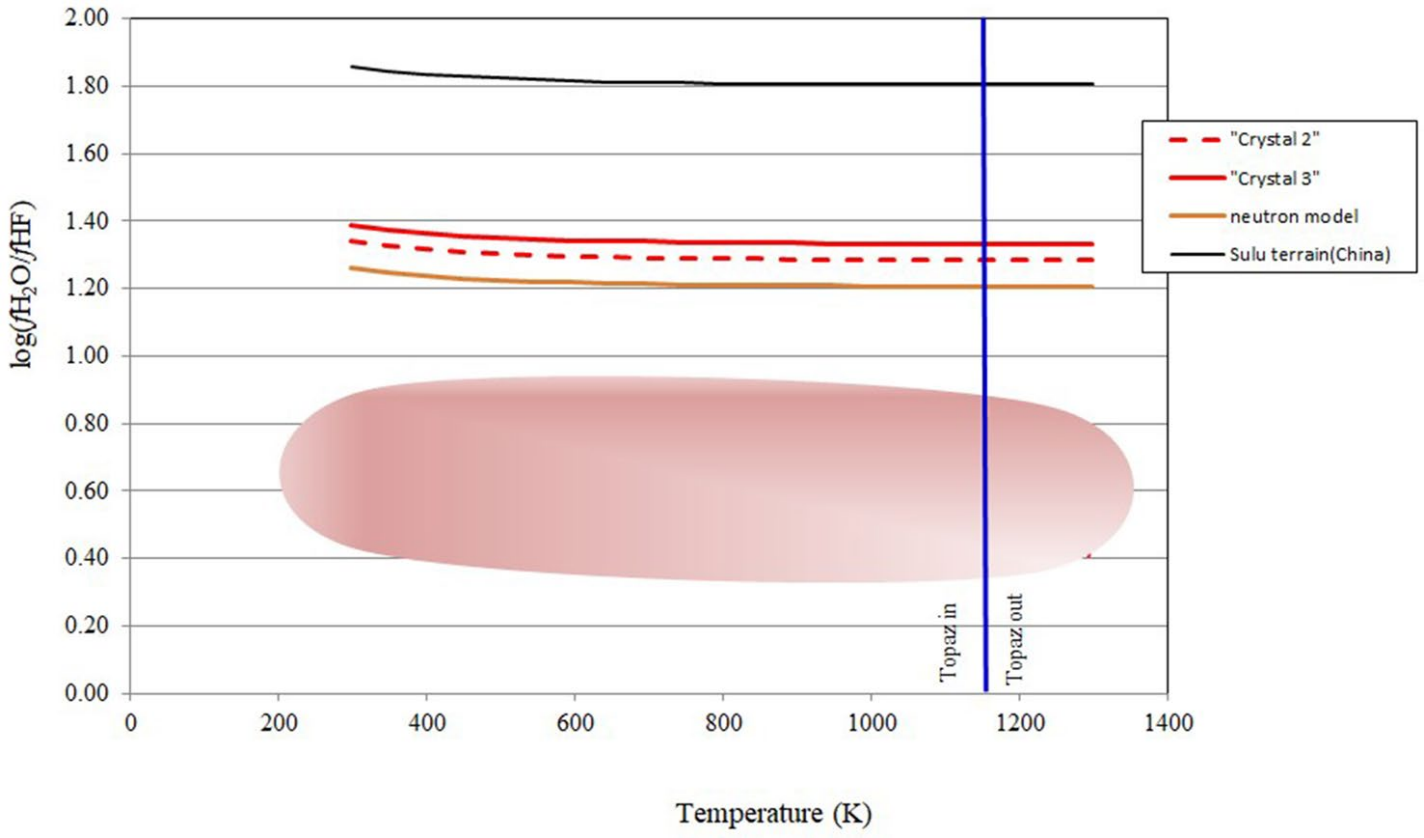
Log fugacity ratios *versus* temperature (K) plot for an inferred (pegmatite) fluid in equilibrium with PadPar topaz. Log(*f*H_2_O/*f*HF)^fluid^ *versus* T show slightly lower fugacity values at increasing temperature. Calculations using chemical analyses (red lines), and neutron diffraction model (yellow line) are reported here. Pink shadow field: log(*f*H_2_O/*f*HF)^fluid^ calculated on topaz sampled in various sites of the Proterozoic Eastern Brazilian Pegmatite province^23–26,56,61^; black line: the log(*f*H_2_O/*f*HF)^fluid^ calculated on the OH- richest natural topaz so far recorded (from quarzites in Sulu terrane, China^1,2^). The potential initial crystallization temperature of topaz in the PadPar pegmatite system is also marked.

Log(*f*H_2_O/*f*HF)^fluid^ slightly decrease at increasing temperature (Fig. 10), at constant topaz composition. It is relevant to observe that the log(*f*H_2_O/*f*HF)^fluid^/temperature curve becomes independent of temperature in the proximity of the suggested limit of topaz stability. Two PadPar topaz crystals in Tables 1 and 2 have similar F–OH occupancy (X_OH_ = 0.474- 0.476) on the hydroxyl site, powdered crystal 2 analysed by neutron diffraction shows a slightly higher fluorine content (X_OH_ = 0.456) with respect to in situ chemical data. This variability is reflected in the calculated (*f*H_2_O/*f*HF)^fluid^ (Fig. 10). For an assumed crystallization temperature of 1170 K the log(*f*H_2_O/*f*HF)^fluid^ is 1.28, 1.33 and 1.21 of fluids in equilibrium with crystal 2, crystal 3 and the neutron model, respectively. Averaging these values, we can estimate that the fluid activity that formed the PadPar topaz has log(*f*H_2_O/*f*H F)^fluid^ ~ 1.27 (0.06). This value is higher with respect to the values calculated by pegmatite topaz sampled in various sites of the Proterozoic Eastern Brazilian Pegmatite province and approaches the Sulu topaz system (Fig. 10). Topaz from quartzite in Sulu terrane that, to the authors’s knowledge; represent the OH- richest natural topaz so far recorded (F = 9.50 − X_OH_ = 0.54), is calculated to be in equilibrium with fluids with log(*f*H_2_O/*f*HF)^fluid^ = 1.80.

The calculated low fluorine activity of the PadPar fluid system in which the mineral is forming, is coherent with the values of hydrothermal fluids (pegmatitic stage) (log (*f*H_2_O/*f*HF)^fluid^ = 1.2–6.5) and differs from pure magmatic (granitic) residual melts [log(*f*H_2_O/*f*HF)^fluid^ < 1]^72,73^.

All together the PadPar topaz type is stabilized by H_2_O saturated fluids with an F content not exceeding 1 wt%^42^. This suggests that among the various topaz-bearing pegmatites of Minas Gerais, the PadPar pegmatite system is a water-dominant fluid lens^61,74,75^.

## Conclusion

We applied a multi analytical strategy to fully characterize the gem quality of coloured topaz from pegmatites of the early Proterozoic Eastern Brazilian Pegmatite Province. The relative simplicity of the topaz chemistry is complicated by the light nature of the major elements forming this mineral (Si, Al, OH and F), therefore gaining chemical-physical information about the crystallization condition was challenging. The successful strategy to combine EDS microanalyses with synchrotron X-Ray and neutron powder diffraction measurements allowed us to accurately determine the mineral structure.

On the basis of neutron diffraction data, the fluorine content is estimated to be ~ 1.03 a.f.u, corresponding to 10.34 wt%, perfectly in agreement with the chemical data (on average 10.0 wt%). The chemical formula is Al_1.92_Si_0.96_O_4.00_F_1.032_OH_0.968_ with X_OH_ = 0.484. Unit cell parameters indicate a positive thermal expansion up to 1010 K, followed by a phase of octahedral expansion regularly counterbalanced by a tetrahedral contraction, up to 1170 K. Above this temperature, both fluorine content and topaz crystallinity decrease, and mullite starts its growth over topaz. This maximum temperature is interpreted as the potential initial crystallization temperature of topaz in the pegmatite fluid system.

The F/OH ratio in this phase is crucial not only for the forming gem process, but also to better understand the circulation of fluids (H_2_O/F) in the forming environment. The fluorine content, expressed as the ratio log(*f*H_2_O/*f*HF)^fluid^, of possible fluids (or H_2_O-F saturated) coexisting with the PadPar topaz, was modelled on the basis of the partitioning of F–(Cl)–OH behaviour between fluorine bearing minerals and late- post magmatic pegmatitic fluids. In doing so, we are confident to conclude that the PadPar fluorine-poor topaz was formed in a lens of fluorine poor/water saturated pegmatite fluids/ in the large early Proterozoic Eastern Brazilian Pegmatite province.

## Supplementary Information

Supplementary Table S1.

Supplementary Table S2.

Supplementary Table S3.
