# Supplementary material for: F/OH ratio in a rare fluorine-poor blue topaz from Padre Paraíso (Minas Gerais, Brazil) to unravel topaz’s ambient of formation
Source: Sci Rep. 2021 Jan 29;11:2666. doi: 10.1038/s41598-021-82045-2 (PMC7846733; doi:10.1038/s41598-021-82045-2)
Supplement: Supplementary file 1 — Supplementary Table S1. [file 41598_2021_82045_MOESM1_ESM.docx]

**F/OH ratio in a rare fluorine-poor blue topaz from Padre Paraíso (Minas Gerais, Brazil) to unravel topaz’s ambient of formation.**

**Precisvalle N.^1^, Martucci A.^1*^, Gigli L.^2^, Plaisier J.R.^2^, Hansen T.C.^3^, Nobre A. G.^4^, Bonadiman C.^1*^**

| **Atom** | **Site** | **298K** | **776K** | **1073K** | **1273K** |
| --- | --- | --- | --- | --- | --- |
| Si | *x*/*a* | 0.3978(2) | 0.39759(27) | 0.39680(31) | 0.39627(32) |
|  | *y*/*b* | 0.94119(12) | 0.94090(12) | 0.94056(14) | 0.94064(14) |
|  | *z*/*c* | 0.25 | 0.25 | 0.25 | 0.25 |
|  | Frac | 1.0 | 1.0 | 1.0 | 1.0 |
|  | Uiso*100 | 0.27(1) | 0.57(1) | 1.07(1) | 2.13(1) |
| Al | *x*/*a* | 0.90419(19) | 0.90330(20) | 0.90339(23) | 0.90338(24) |
|  | *y*/*b* | 0.13114(9) | 0.13130(9) | 0.13123(11) | 0.13160(11) |
|  | *z*/*c* | 0.08234(10) | 0.08214(11) | 0.08216(12) | 0.08232(13) |
|  | Frac | 1.0 | 1.0 | 1.0 | 1.0 |
|  | Uiso*100 | 0.29(1) | 0.59(1) | 1.09(1) | 2.18(1) |
| O1 | *x*/*a* | 0.79370(43) | 0.79620(44) | 0.79827(51) | 0.79747(51) |
|  | *y*/*b* | 0.53196(24) | 0.53234(25) | 0.53243(29) | 0.53238(28) |
|  | *z*/*c* | 0.25 | 0.25 | 0.25 | 0.25 |
|  | Frac | 1.0 | 1.0 | 1.0 | 1.0 |
|  | Uiso*100 | 0.47(1) | 0.76(1) | 2.07(1) | 4.04(1) |
| O2 | *x*/*a* | 0.45694(45) | 0.45808(47) | 0.45899(54) | 0.45756(54) |
|  | *y*/*b* | 0.75633(26) | 0.75734(27) | 0.75731(31) | 0.75713(31) |
|  | *z*/*c* | 0.25 | 0.25 | 0.25 | 0.25 |
|  | Frac | 1.0 | 1.0 | 1.0 | 1.0 |
|  | Uiso*100 | 0.46(1) | 0.76(1) | 2.06(1) | 4.15(1) |
| O3 | *x*/*a* | 0.79002(29) | 0.79151(30) | 0.79179(35) | 0.79160(35) |
|  | *y*/*b* | 0.01115(16) | 0.01198(17) | 0.01224(19) | 0.01180(19) |
|  | *z*/*c* | 0.90736(19) | 0.90650(19) | 0.90580(22) | 0.90621(22) |
|  | Frac | 1.0 | 1.0 | 1.0 | 1.0 |
|  | Uiso*100 | 0.43(1) | 0.73(1) | 2.03(1) | 4.12(1) |
| F | *x*/*a* | 0.90026(32) | 0.90067(32) | 0.9001(4) | 0.9012(4) |
|  | *y*/*b* | 0.75290(17) | 0.75264(15) | 0.75273(18) | 0.75301(18) |
|  | *z*/*c* | 0.05731(17) | 0.05686(16) | 0.05612(18) | 0.05679(19) |
|  | Uiso*100 | 0.41(1) | 0.71(1) | 2.01(1) | 4.02(1) |
| O4 | *x*/*a* | 0.90026(32) | 0.90067(32) | 0.9001(4) | 0.9012(4) |
|  | *y*/*b* | 0.75290(17) | 0.75264(15) | 0.75273(18) | 0.75301(18) |
|  | *z*/*c* | 0.05731(17) | 0.05686(16) | 0.05612(18) | 0.05679(19) |
|  | Uiso | 0.41(1) | 0.71(1) | 2.01(1) | 4.02(1) |
| H | *x*/*a* | 0.0318(28) | 0.022(13) | 0.030(14) | 0.024(14) |
|  | *y*/*b* | 0.7154(30) | 0.703(9) | 0.715(11) | 0.711(11) |
|  | *z*/*c* | 0.1352(26) | 0.123(9) | 0.117(11) | 0.127(10) |
|  | Uiso*100 | 0.69(1) | 0.90(1) | 4.09(1) | 6.09(1) |

Supplementary table S1. Atomic coordinates, fractions and thermal parameters for synchrotron data diffraction at 298, 776 and 1273K rom *in situ* time resolved data.
