# Supplementary material for: F/OH ratio in a rare fluorine-poor blue topaz from Padre Paraíso (Minas Gerais, Brazil) to unravel topaz’s ambient of formation
Source: Sci Rep. 2021 Jan 29;11:2666. doi: 10.1038/s41598-021-82045-2 (PMC7846733; doi:10.1038/s41598-021-82045-2)
Supplement: Supplementary file 2 — Supplementary Table S2. [file 41598_2021_82045_MOESM2_ESM.docx]

**F/OH ratio in a rare fluorine-poor blue topaz from Padre Paraíso (Minas Gerais, Brazil) to unravel topaz’s ambient of formation.**

**Precisvalle N.^1^, Martucci A.^1*^, Gigli L.^2^, Plaisier J.R.^2^, Hansen T.C.^3^, Nobre A. G.^4^, Bonadiman C.^1*^**

Supplementary table S2. Atomic coordinates, fractions and thermal parameters for neutron data diffraction at 298, 776 and 1273K rom *in situ* time resolved data.

| ***Atom*** | ***Site*** | ***298K*** | ***776K*** | ***1073K*** | ***1273K*** |
| --- | --- | --- | --- | --- | --- |
| Si | *x*/*a* | 0.3815(21) | 0.3899(20) | 0.3892(20) | 0.3823(28) |
|  | *y*/*b* | 0.9453(10) | 0.9432(10) | 0.9427(11) | 0.9400(15) |
|  | *z*/*c* | 0.25 | 0.25 | 0.25 | 0.25 |
|  | Occ | 1.0 | 1.0 | 1.0 | 1.0 |
|  | Uiso*100 | 0.21(1) | 0.58(1) | 1.10(1) | 2.37(5) |
| Al | *x*/*a* | 0.8936 (20) | 0.8984(18) | 0.8967(19) | 0.8945(33) |
|  | *y*/*b* | 0.1324(8) | 0.1313(8) | 0.1318(9) | 0.1288(14) |
|  | *z*/*c* | 0.0798(11) | 0.0807(11) | 0.0810(11) | 0.0837(17) |
|  | Occ | 1.0 | 1.0 | 1.0 | 1.0 |
|  | Uiso*100 | 0.31(1) | 0.52(1) | 1.80(1) | 2.12(5) |
| O1 | *x*/*a* | 0.8131(15) | 0.8032(14) | 0.8035(14) | 0.8061(24) |
|  | *y*/*b* | 0.5320(8) | 0.5344(7) | 0.5337(8) | 0.5273(19) |
|  | *z*/*c* | 0.25 | 0.25 | 0.25 | 0.25 |
|  | Occ | 1.0 | 1.0 | 1.0 | 1.0 |
|  | Uiso | 0.47(1) | 0.72(1) | 2.60(1) | 4.16(3) |
| O2 | *x*/*a* | 0.4612(15) | 0.4572(15) | 0.4568(15) | 0.4757(34) |
|  | *y*/*b* | 0.7552(11) | 0.7579(10) | 0.7585(11) | 0.7593(18) |
|  | *z*/*c* | 0.25 | 0.25 | 0.25 | 0.25 |
|  | Occ | 1.0 | 1.0 | 1.0 | 1.0 |
|  | Uiso*100 | 0.46(1) | 0.76(1) | 2.10(1) | 4.02(3) |
| O3 | *x*/*a* | 0.7926(11) | 0.7907(10) | 0.7914(11) | 0.8055(18) |
|  | *y*/*b* | 0.0127(5) | 0.0110(5) | 0.0101(6) | 0.0166(13) |
|  | *z*/*c* | 0.9089(6) | 0.9091(6) | 0.9078(6) | 0.9092(13) |
|  | Occ | 1.0 | 1.0 | 1.0 | 1.0 |
|  | Uiso*100 | 0.43(1) | 0.74(1) | 2.08(1) | 4.02(3) |
| F | *x*/*a* | 0.9055(11) | 0.8963(11) | 0.8951(11) | 0.8977(26) |
|  | *y*/*b* | 0.7505(7) | 0.7547(7) | 0.7538(8) | 0.7583(12) |
|  | *z*/*c* | 0.0552(5) | 0.0545(5) | 0.0538(5) | 0.0528(13) |
|  | Occ | 0.516(2) | 0.5135(2) | 0.5190(1) | 0.3150(1) |
|  | Uiso | 0.41(1) | 0.71(1) | 2.15(1) | 4.25(3) |
| O4 | *x*/*a* | 0.9055(11) | 0.8963(11) | 0.8951(11) | 0.8977(26) |
|  | *y*/*b* | 0.7505(7) | 0.7547(7) | 0.7538(8) | 0.7583(12) |
|  | *z*/*c* | 0.0552(5) | 0.0545(5) | 0.0538(5) | 0.0528(13) |
|  | Occ | 0.48(2) | 0.4833(2) | 0.4840(1) | 0.4816(2) |
|  | Uiso*100 | 0.411(1) | 0.71(1) | 2.15(1) | 4.25(3) |
| H | *x*/*a* | 0.3026(15) | -0.035(9) | 0.001(20) | 0.0338(8) |
|  | *y*/*b* | 0.7325(11) | ­0.774(6) | 0.748(16) | 0.7177(9) |
|  | *z*/*c* | 0.1476(11) | 0.1609(20) | 0.153(7) | 0.1290(7) |
|  | Occ | 0.484(2) | 0.4833(2) | 0.4840(1) | 0.4816(2) |
|  | Uiso*100 | 0.59(3) | 0.96(3) | 3.446(3) | 5.53(7) |
