# Supplementary material for: F/OH ratio in a rare fluorine-poor blue topaz from Padre Paraíso (Minas Gerais, Brazil) to unravel topaz’s ambient of formation
Source: Sci Rep. 2021 Jan 29;11:2666. doi: 10.1038/s41598-021-82045-2 (PMC7846733; doi:10.1038/s41598-021-82045-2)
Supplement: Supplementary file 3 — Supplementary Table S3. [file 41598_2021_82045_MOESM3_ESM.docx]

**F/OH ratio in a rare fluorine-poor blue topaz from Padre Paraíso (Minas Gerais, Brazil) to unravel topaz’s ambient of formation.**

**Precisvalle N.^1^, Martucci A.^1*^, Gigli L.^2^, Plaisier J.R.^2^, Hansen T.C.^3^, Nobre A. G.^4^, Bonadiman C.^1*^**

Supplementary table S3. Major elements composition of Padre Paraíso topaz from EDS analyses and neutron diffraction.

|  | Padre Paraíso | |  |  |  |  |  |  |  |  |  |  |  |  |
| --- | --- | --- | --- | --- | --- | --- | --- | --- | --- | --- | --- | --- | --- | --- |
| topaz | crystal 1 |  |  |  |  |  | crystal 2 |  |  |  |  |  | (neutron) |  |
| spots | 01 core | 02 core | 03 core | 04 core | 05 core |  | 01 core | 02 core | 03 core | 04 core | 05 core |  |  |  |
|  |  |  |  |  |  |  |  |  |  |  |  |  |  |  |
| SiO_2_ (wt.) | 32.87 | 34.38 | 34.40 | 33.20 | 33.05 |  | 33.07 | 33.05 | 34.13 | 32.94 | 32.69 |  |  |  |
| Al_2_O_3_ | 57.11 | 56.61 | 56.45 | 54.56 | 57.36 |  | 57.92 | 54.90 | 57.85 | 56.43 | 57.22 |  |  |  |
| CaO | 0.00 | 0.00 | 0.01 | 0.00 | 0.00 |  | 0.00 | 0.00 | 0.03 | 0.00 | 0.00 |  |  |  |
| Na_2_O | 0.01 | 0.00 | 0.00 | 0.00 | 0.02 |  | 0.00 | 0.00 | 0.00 | 0.00 | 0.01 |  |  |  |
| F | 10.03 | 9.01 | 9.15 | 12.24 | 9.58 |  | 9.01 | 12.05 | 8.02 | 10.62 | 10.09 |  |  |  |
| O=F, Cl | 4.00 | 3.61 | 3.65 | 4.85 | 3.83 |  | 3.61 | 4.78 | 3.21 | 4.23 | 4.02 |  |  |  |
| Total | 96.00 | 96.39 | 96.35 | 95.15 | 96.17 |  | 96.39 | 95.22 | 96.79 | 95.77 | 95.98 |  |  |  |
|  |  |  |  |  |  |  |  |  |  |  |  |  |  |  |
| a.f.u. |  |  |  |  |  |  |  |  |  |  |  |  |  |  |
| Si | 0.984 | 1.020 | 1.022 | 1.022 | 0.985 |  | 0.979 | 1.014 | 1.001 | 0.994 | 0.979 |  |  |  |
| Al | 2.016 | 1.980 | 1.978 | 1.978 | 2.015 |  | 2.021 | 1.986 | 1.999 | 2.006 | 2.021 |  |  |  |
| Ca | 0.000 | 0.000 | 0.000 | 0.000 | 0.000 |  | 0.000 | 0.000 | 0.000 | 0.000 | 0.000 |  |  |  |
| Na | 0.000 | 0.000 | 0.000 | 0.000 | 0.000 |  | 0.000 | 0.000 | 0.000 | 0.000 | 0.000 |  |  |  |
|  |  |  |  |  |  |  |  |  |  |  |  |  |  |  |
| F | 1.055 | 0.949 | 0.963 | 1.288 | 1.009 |  | 0.949 | 1.269 | 0.845 | 1.118 | 1.062 |  | **1.034** |  |
| OH° | 0.945 | 1.051 | 1.037 | 0.712 | 0.991 |  | 1.051 | 0.731 | 1.155 | 0.882 | 0.938 |  | **0.966** |  |
|  |  |  |  |  |  |  |  |  |  |  |  |  |  |  |
| X_OH_ | 0.472 | 0.526 | 0.518 | 0.356 | 0.496 |  | 0.526 | 0.366 | 0.578 | 0.441 | 0.469 |  | **0.483** |  |
|  |  |  |  |  |  |  |  |  |  |  |  |  |  |  |
| OH° = by stoichiometric formula; X_OH_ =OH/(F+OH); atomic formula units (a.f.u): normalized to 3 cations and F+OH = 2 a.f.u. from neutron diffraction. | | | | | | | | | | | | | | |
|  |  |  |  |  |  |  |  |  |  |  |  |  |  |  |
